# Prospecting endophytic fungal assemblage of *Digitalis lanata* Ehrh. (foxglove) as a novel source of digoxin: a cardiac glycoside

**DOI:** 10.1007/s13205-012-0106-0

**Published:** 2012-12-15

**Authors:** Sanjana Kaul, Maroof Ahmed, Khalid Zargar, Pooja Sharma, Manoj K. Dhar

**Affiliations:** School of Biotechnology, University of Jammu, Jammu, 180006 India

**Keywords:** Endophytes, Secondary metabolites, Glycosides, *Digitalis*, Digoxin, HPLC

## Abstract

Endophytes, the chemical synthesizers inside plants, are the microorganisms having mutualistic relationship with the host plant. They can be used by plants for defense in addition to the production of a wide variety of beneficial bioactive secondary metabolites. There are reports that microbial endophytes mimic the bioactive compounds as produced by the plant itself thus making them a promising source of novel compounds. During the present study, endophytes were isolated from the symptomless leaves and stem of the angiosperm, *Digitalis lanata* (foxglove). *Digitalis lanata* belongs to the family Plantaginaceae and is an important medicinal plant known for the production of an important glycoside, digoxin having valuable medicinal importance. Glycosides from *Digitalis* have been reported to be cardiotonic and are widely used in the treatment of various heart conditions namely atrial fibrillation, atrial flutter, heart failure, etc. Endophytic fungi were isolated from *Digitalis* to screen them for such glycosides as have been found in the plant itself. A total of 35 fungal endophytes were isolated and screened for the production of secondary metabolites. After preliminary analysis by thin layer chromatography for the presence of bioactive compounds, crude extracts of five fungal cultures were selected for HPLC. HPLC chromatograms revealed the production of glycoside digoxin from the five selected endophytic cultures, thus providing a novel, alternative and eco-friendly source for the production of such a pharmaceutically important and valuable drug.

## Introduction

Plants have been known to act as host to various endophytic microorganisms known to produce plethora of substances of potential use to modern medicine, agriculture and pharmaceutical industry (Owen and Hundley 2004). Endophytes usually have a mutualistic relationship with that of the host and are responsible for the adaptation of plants to abiotic stress such as drought, light, metals and biotic stress like herbivory, insects and pathogens (Arnold et al. 2003; Carroll 1988; Clay 1988; Faeth 2002). Endophytic fungi have been found in a diverse array of plant species examined so far including algae, mosses, conifers, angiosperms, and gymnosperms (U’ren et al. 2010). Endophytic fungi inhabiting the foliage of woody plants are far less studied than endophytes of grasses (Arnold and Herre 2003). Endophytes include fungi, bacteria and actinomycetes, although the most frequently isolated endophytes are the fungi. Endophytic fungi are considered as an outstanding source of bioactive natural products because there are many occupying millions of unique biological niches and growing in different types of environment (Strobel and Daisy 2003). Plants infected with fungal endophytes are often healthier than endophyte free ones (Waller et al. 2005). This effect may be due to the production of phytohormones such as indole 3-acetic acid (IAA), cytokines and plant growth promoting substances (Tan and Zou 2001). There are also quite a large number of biologically active compounds which have been isolated from endophytic fungi e.g., novel compounds like anticancer drugs, antibiotics and immunosuppressive compounds (Strobel and Daisy 2003). Various bioactive natural products derived from endophytic fungi belong to diverse structural groups including glycosides, flavonoids, terpenoids, steroids, xanthones, chinones, phenols, isocoumarins, benzopyrones, tetralones, etc. (Tan and Zou 2001). The presence of endophytes in plants stimulates the production of secondary metabolites in plants with a diverse range of biological activities (Petrini et al. 2006). Some of the endophytic microorganisms have been found to produce the same secondary metabolites as that of the plant thus making them a promising source of novel compounds. For example, the discovery of the world’s first billion dollar anti-cancer drug paclitaxel (taxol) from *Pestalotiopsis microspora*, an endophytic fungus that colonizes the Himalayan yew tree *Taxuswallichiana* (Strobel et al. 1996; Gangadevi et al. 2008); *Muscodor albus* produces volatile compounds to inhibit and kill a wide spectrum of fungi and bacteria (Strobel et al. 2001); an anticancer compound camptothecin has been reported from *Fusariumsolani* endophytic in *Camptotheca acuminata* (Kusari et al. 2009). Likewise, a number of anticancer compounds have been isolated from fungal endophytes (Kharwar et al. 2011). An insect repellent, azadirachtin (triterpenoid) has been reported from the endophytic fungus *Eupenicillium parvum* inhabiting *Azadirachta indica.* (Kusari et al. 2012a). Wang et al. (2012) isolated new antimicrobial compounds produced by *Phoma sp.* endophytic in *Arisaema erubescens.* Other important secondary metabolites isolated from endophytes include ergoflavin, griseofulvin, hypericin, citrinin, pestacin, methyl eugenol, 2-phenyl ethanol, etc. (Joseph and Priya 2011; Kaul et al. 2008; Wani et al. 2010). Kharwar et al. (2012) studied the diversity, distribution and community structure of endophytic fungi from *Cinnamomum camphora* and also evaluated their antimicrobial potential. Even much more significant research has been done on various aspects of endophytic fungi including diversity, mutualistic paradigms, chemical ecology, production of bioactive metabolites and various other industrial applications (Kaul et al. 2012; Kusari et al. 2012b, c, d; Kusari and Spiteller 2011). Thus, it becomes obvious that an enormous potential for new drug discovery in this field holds exciting promise. This is evidenced by the discovery of a wide range of products and microorganisms that present potential for new drug discovery and consequently, it motivated us to investigate the secondary metabolism of endophytic fungi in a medicinal plant with an aim to find the fungal strain able to produce structurally novel and biologically active secondary metabolites.

*Digitalis* (foxglove), an ornamental angiosperm of about 20 species of herbaceous perennials, shrubs and biennials has been reported to produce cardiotonic drugs and is widely used in the treatment of various heart conditions namely atrial fibrillation, atrial flutter and, sometimes heart failure that cannot be controlled by other medications. It is a powerful diuretic and provides a valuable remedy in dropsy. It has also been employed in the treatment of internal hemorrhage, inflammatory diseases, delirium tremens, epilepsy, acute mania and various other diseases with real or supposed benefit. It is used to increase cardiac contractility (it is a positive inotrope) and as an antiarrhythmic agent to control the heart rate, particularly in the irregular (and often fast) atrial fibrillation (Ahmed et al. 2012; Fatima et al. 2009).

In the present study, efforts have been made to isolate endophytic fungi inhabiting *Digitalis lanata*. The isolated endophytic fungal assemblage was processed and evaluated for the production of compounds particularly glycosides similar to those present in the plant itself.

## Materials and methods

### Source of endophytic fungi

The medicinal plant i.e., *Digitalis lanata* selected for the present study was collected from its natural habitat, Gulmarg (altitude 2,730 m; District Baramulla) Jammu and Kashmir State, India. The samples of the plant material were collected and brought to laboratory in pre-sterilized polythene bags and kept at 4 °C till immediate processing.

### Isolation of endophytic fungi

Endophytic fungal isolation was carried out under aseptic conditions. Different parts of the wooly foxglove (*Digitalis lanata*) such as stem cuttings and leaves were used for the isolation of fungal endophytes (Bacon 1988; Ahmed et al. 2012). The collected plant material used for the isolation was first surface sterilized following the method of Santos et al. (2003) with slight modifications. Plant material was first cleaned by washing several times under running tap water and then cut into small segments. Surface sterilization was performed by sequentially rinsing the plant material with 70 % ethanol (C_2_H_5_OH) for 30 s, then with 0.01 % mercuric chloride (HgCl_2_) for 5 min followed by 0.5 % working concentration of available sodium hypochlorite 4 % solution (NaOCl) for 2–3 min and finally with sterile distilled water 2–3 times. It was then dried in between the folds of sterile filter paper. After proper drying, leaves and stem were cut into smaller segments, each segment was placed in a Petri dish plated with potato dextrose agar (PDA) medium supplemented with chloramphenicol (100 mg/ml). A total of 120 explants were inoculated on 20 PDA-plates (6 explants per plate). All the plates were incubated at 26 °C to promote fungal growth and sporulation. The plates were regularly monitored for any mycelial growth. On observing the mycelial growth sub-culturing of the fungi was done. Each fungal culture was checked for purity and transferred to another agar plate using the hyphal tips. The purified fungal isolates were transferred separately to PDA slants, accessioned and maintained at 4 °C. Appropriate controls were also set up with media-plated plates without any material.

### Extraction of secondary metabolites

Secondary metabolites were extracted from the plant material as well as from the fungal isolates.

### Plant material

Extraction of secondary metabolites was done by Soxhlet extraction method. Leaves of *Digitalislanata* were properly washed, air dried and ground to fine powder. Ten grams of this powder was placed in Soxhlet extractor and the assembly was run using hexane for 6–8 h. After this, solvent was changed and the Soxhlet was run with 50 % methanol (CH_3_OH) for 18–20 h. Crude extract so obtained was concentrated in a water bath and then hydrolyzed in 10 % HCl for 1 h. The contents were then subsequently separated with chloroform in a separating funnel. Chloroform fractions were collected, concentrated and checked on preparative TLC plates.

### Selected endophytic fungal isolates

Extraction of secondary metabolites from fungal endophytes was done by Soxhlet extraction method. A total of 35 fungal endophytes were isolated and screened for the production of secondary metabolites. Freshly grown fungal isolates were cultured in 150 ml of potato dextrose production medium in a 250 ml conical flask. The flasks were incubated at 27 °C in shaking conditions at 140 rpm. Maximum growth and optimum production were evaluated on the 12th day of incubation. The protocol was standardized for isolation of secondary metabolites from fungal endophytes. The fungal biomass was then filtered through muslin cloth and allowed to dry on a filter paper. The dried fungal biomass was then extracted with n-hexane in the Soxhlet extractor assembly for 8–10 h. After extraction with n-hexane the Soxhlet was run with 50 % methanol for complete extraction of metabolites for 18–20 h from fungal biomass. The extract was then concentrated in a water bath followed by hydrolysis with 10 % HCl. Separation was carried out with chloroform in a separating funnel. Chloroform fractions were collected, concentrated and checked by thin layer chromatography (TLC).

TLC of all the samples was performed on silica gel plates with the solvent system ethyl-acetate:methanol:water in the ratio of (81:11:8) v/v. The compounds were detected by spraying 4 % sulfuric acid as the developing reagent onto the TLC plates. The compounds appeared as specific spots after baking the TLC plates for 10 min at 110 °C.

### High performance liquid chromatography (HPLC) analysis

HPLC was performed to confirm and purify the compounds in the samples. The HPLC system used in the present study consisted of an Agilent series 1100 equipped with a binary pump, an auto sampler an automatic degasser, an automatic thermostatic column oven, diode array detector and a computer with chemstation software for data analysis. The HPLC conditions used were as follows: Column used Merck RP-18 (125 mm × 4.6 mm: 5 μm), mobile phase (80:20) acetonitrile:water, flow rate 100 μl/min, and wavelength 210 nm.

## Results and discussion

A total of 35 fungal endophytes were isolated from asymptomatic parts such as leaves and stem of *Digitalis lanata*. A total of 120 explants of both leaf and stem segments were inoculated on 20 PDA-plates (6-explants per plate). Nineteen fungal endophytes from leaves and sixteen fungal endophytes from the stem were isolated. The isolated endophytic fungi were then identified on the basis of morphological characters such as growth pattern, hyphae, color of colony, surface texture, margin character and characteristics of the spore (Barnett and Hunter 1956). It was found that among the fungal assemblage, frequency of *Alternaria*, *Penicillium* and *Aspergillus* species was maximum when compared to other genera, while few of them could not be identified due to lack of either sporulation or other significant characters of identification. Depending on the frequency of the species, they were taken up for further studies. The isolated fungal endophytes were further studied for glycoside production. They were subjected to liquid shake fermentation for the production of secondary metabolites. Secondary metabolites from endophytes have been reported to include growth hormones, antimicrobial or anticancer substances (Petrini et al. 1992; Huang et al. 2001). There are reports on secondary metabolites being potential drugs for treatment of newly developing diseases in humans and also plants and animals (Strobel and Daisy 2003). Endophytes from the medicinal plants are a treasure hunt for the production of bioactive metabolites (Kaul et al. 2012).

In our study, we used the plant extract along with the standard for comparative analysis. Thin layer chromatography screening revealed that only five endophytic isolates accessioned as DL-3, DL-11, DL-12, DS-20 and DS-22 produced the spots corresponding to the relevant spot of the standard and plant extract. Thus, out of the total fungal assemblage consisting of 35 isolated endophytic fungi, only five potent isolates were selected for HPLC-analysis after the preliminary screening by TLC.

**Fig. 1:**
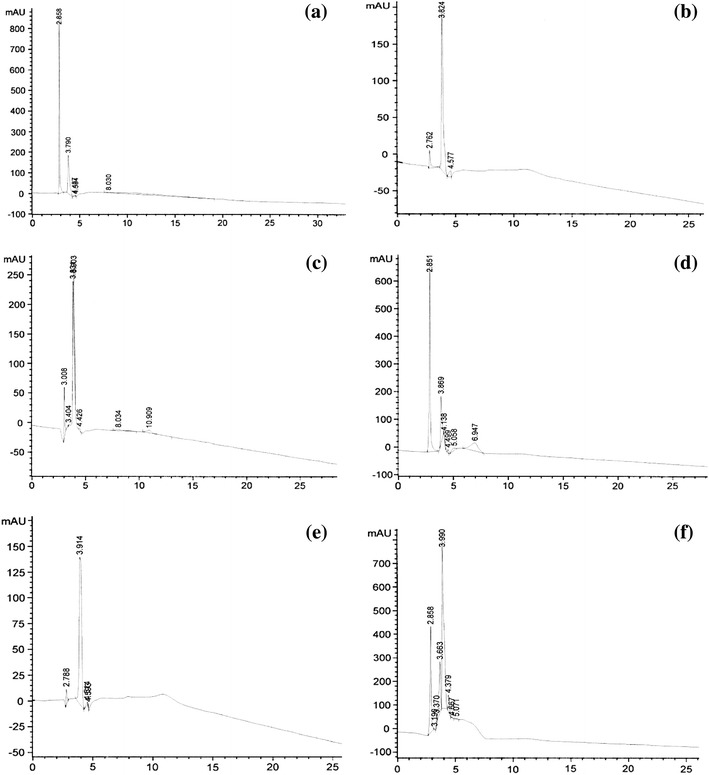
HPLC-chromatograms of plant and endophytic fungal extracts exhibiting various peaks. **a** Chromatogram of the crude plant extract (*Digitalislanata*) indicating various peaks. Peak at the retention time of 3.790 min corresponds to digoxin. **b** Chromatogram of the accession DL-3 indicating various peaks. Peak at the retention time of 3.824 min corresponds to digoxin. **c** Chromatogram of the accession DL-12 indicating various peaks. Peak at the retention time of 3.831 min corresponds to digoxin. **d** Chromatogram of the accession DS-20 indicating various peaks. Peak at the retention time of 3.869 min corresponds to digoxin. **e** Chromatogram of the accession DL-11 indicating various peaks. Peak at the retention time of 3.914 min corresponds to digoxin. **f** Chromatogram of the accession DS-22 indicating various peaks. Peak at the retention time of 3.663 min corresponds to digoxin

From the chromatograms, it could be observed that the plant extract (Fig. 1a) along with the endophytic fungal accessions (Fig. 1b–f) eluted different compounds at different retention times as are exhibited by various peaks. Here it could be seen that fungal endophytes mimic some of the compounds as produced by the plant itself. This was revealed by retention times which exactly/nearly correspond with the retention times of various peaks shown by the plant extract. Most prominent peaks from all the fungal accessions at the retention times of 3.824, 3.914, 3.831, 3.869 and 3.663 min, approximately, correspond to one of the compounds eluted by the plant extract at a retention time of 3.790 min which was also the retention time of standard (digoxin) under the same HPLC conditions. Thus, it can be concluded that the main chemical constituent of *Digitalis lanata* i.e., the glycosidic compound digoxin was produced by the five fungal endophytes under investigation that were isolated from this plant. Apart from this, some other compounds from the fungal isolates also correspond with the rest of the compounds eluted from the plant extract i.e., the compound from isolate DS-22 at the retention time of 2.858 min exactly corresponds to one of the peaks from the plant extract and nearly with one of the peaks from all other fungal isolates. Likewise, rest of the peaks from the plant extract nearly corresponds to some of the peaks from the endophytic fungal accessions. Thereby, confirming that the fungal endophytes do mimic the bioactive compounds as produced by the plant itself (digoxin in this case). Thus, providing a new hope for the production of novel metabolites through alternative means that even being eco-friendly.

Natural products from fungal endophytes have a broad spectrum of biological activity and they can be grouped into several categories including; alkaloids, steroids, terpenoids, isocoumarins, quinones, phenyl propanoids and lignans, aliphatic metabolites, lactones, etc. (Zhang et al. 2006). Puri et al. (2005), isolated a novel Camptothecin producing endophytic fungus *Entrophosphora inferquens* from an important Indian medicinal plant *Nothapodytes foetida*. *E.inferquens* synthesizes camptothecin which showed potential immunomodulatory activity. Chen et al. (2007) isolated an endophytic fungus *Pencilliumthomi* from the roots of *Bruguiera gymnorhiza.* The separation of endophytic fungus from the root led to the isolation of a new compound 4′,5 dihydroxy-2,3 dimethoxy 4(-hydroxy propyl)-biphenyl along with 11 known compounds. Their effects against three human cell lines were investigated. In the series of useful bioactive compounds from plants, two important compounds are digoxin and digitoxin. These are plant glycosides (Cardiac glycosides) which are widely used in medicine. These glycosides are produced from the medicinal plant *Digitalis lanata.Digitalis* glycosides consist of a steroid nucleus, an unsaturated lactone ring and a sugar stuck on to the lactone ring. The sugar determines solubility, half life, etc. The main chemical constituent of *Digitalis lanata* is Digoxin. Digoxin has an extra OH group making it more polar (less GI absorption, reduced protein binding and increased urinary excretion related to digitoxin). Digoxin is eliminated chiefly by the liver/kidneys. It exists as odorless white crystals that melt with decomposition above 230 °C. The drug is practically insoluble in water and in ether; slightly soluble in diluted (50 %) alcohol and in chloroform; and freely soluble in pyridine.

Kwon et al. (2010) detected cardiac glycosides from the leaves of *Digitalis* by reversed phase HPLC-pulsed amperometric detection. Further, the content of the cardiotonic glycosides from *Digitalis* was determined by Alonso et al. (2009). A microbial source for the production of this glycoside would really be beneficial.

Therefore, keeping in view the pharmaceutical applications of digoxin, these glycoside producing potent strains of endophytic fungi comprising a fungal assemblage could be further worked out on a pilot scale for process optimization and scale-up studies. This study signifies a novel microbial source for the digoxin-like glycosides.

## Conclusions

Endophytic fungi have recently received more attention as they can sometimes produce bioactive compounds analogous to their hosts. The mechanism is still not clear but it is assumed that the genes which control the synthesis of bioactive compounds might be transferred between endophytic fungi and the host during long time symbiosis. We can say that the endophytic fungi could be a reliable source for pharmaceutically and industrially important compounds that can be used in the treatment of various life-threatening diseases along with various industrial applications. Applications of endophytes in pharmaceutical industries include cost effective drug production, endophytes as drug source help us to conserve biodiversity and drug resistance as they are an alternate source of drugs.
